# Validation of the *β-amy1* Transcription Profiling Assay and Selection of Reference Genes Suited for a RT-qPCR Assay in Developing Barley Caryopsis

**DOI:** 10.1371/journal.pone.0041886

**Published:** 2012-07-31

**Authors:** Jaroslava Ovesná, Ladislav Kučera, Kateřina Vaculová, Kamila Štrymplová, Ilona Svobodová, Luigi Milella

**Affiliations:** 1 Department of Molecular Biology, Crop Research Institute, Prague, Czech Republic; 2 Agrotests phyto Ltd., Kroměříž, Czech Republic; 3 Department of Biology-Basilicata University, Potenza, Italy; Kyushu Institute of Technology, Japan

## Abstract

Reverse transcription coupled with real-time quantitative PCR (RT-qPCR) is a frequently used method for gene expression profiling. Reference genes (RGs) are commonly employed to normalize gene expression data. A limited information exist on the gene expression and profiling in developing barley caryopsis. Expression stability was assessed by measuring the cycle threshold (Ct) range and applying both the GeNorm (pair-wise comparison of geometric means) and Normfinder (model-based approach) principles for the calculation. Here, we have identified a set of four RGs suitable for studying gene expression in the developing barley caryopsis. These encode the proteins GAPDH, HSP90, HSP70 and ubiquitin. We found a correlation between the frequency of occurrence of a transcript *in silico* and its suitability as an RG. This set of RGs was tested by comparing the normalized level of β-amylase (*β-amy1*) transcript with directly measured quantities of the BMY1 gene product in the developing barley caryopsis. This panel of genes could be used for other gene expression studies, as well as to optimize *β-amy1* analysis for study of the impact of *β-amy1* expression upon barley end-use quality.

## Introduction

Gene expression analysis is a major focus of current biological research and large data sets continue to be generated from the application of various analytical platforms [1], [2]. The direct quantification of a given protein present in a tissue or its level of biological activity can be technically challenging, but transcript levels are relatively straightforward to obtain by taking advantage of a number of possible technology platforms [3]. RT-qPCR is frequently exploited to measure gene expression level or to validate results of DNA arrays assays. However, Bustin et al. [4] have emphasized that some of the apparent differences that emerge from many transcriptomic analyses are artefactual, due to uncontrolled variation in, among other things, sample preparation, nucleic acid isolation, cDNA synthesis and PCR amplification. These factors contribute to a variable extent from poor reproducibility to inaccurate data [5], [6], [7]. Thus, it is important for RT-qPCR data be normalized before any comparisons are attempted between independent samples or experiments. Normalization is typically based on either the expression of a constitutively expressed gene or total RNA content. The limitations of the latter are understood and its precision is highly dependent on the accurate quantification of the RNA content of the sample [8], [9]. The former strategy can be extended to two or more RGs and various methods have been established to use RGs expression levels to correct raw expression data [10].

Numerous studies have been published describing appropriate reference gene for certain plant species, tissue and/or environmental conditions [11]–[15]. Studies have also been published focusing on cereals including wheat and barley where the authors have aimed to find either universal normalization genes across related species or environmental conditions [7], [15], [16] or specific stress and tissue [14]. Although published data could be directly taken, necessity of careful selection and verification of housekeeping genes for individual tissue and certain experimental conditions are recommended [16]–[19]; otherwise normalization could lead to inaccurate conclusions. Some authors encourage seeking for accurate genes for normalization not only for animal but also for plant species [7], [14], [20]. Gimenéz et al. [16] have stressed that the choice and optimal number of reference genes must be experimentally determined.

In this study we aimed to establish a panel of RGs that can be used to quantify the expression of genes involved in determining the quality of barley grain. In addition, we report a qRT-PCR assay that allows for the expression profiling of the *β-amy1* gene. “Endosperm-specific” *β-amy1* is one of the four barley malt enzymes involved in fermentable sugar production during mashing. Of the four malt enzymes, *β-amy1* best correlates with diastatic power, a measurement of total amylolytic activity and an important determinant of malt quality [21]. We report precise variability of individual steps of the assay considering the recommendations proposed by Vandesompele et al. [22].

## Materials and Methods

### Plant Material, RNA Extraction and cDNA Synthesis

The seeds of three spring barley cultivars were obtained from the Agricultural Research Institute Kromeriz: the spring barley Jersey seeds were used for selection of RGs and the seeds of other two spring barley malting cultivars were used for validation of the developed *β-amy1* assay. All three genotypes possess alleles with intermediate thermostability as shown in previous work [23]. Developing caryopses were collected at 5, 10, 15, 20 and 25 days after anthesis in two successive years. The embryos were dissected into RNAlater® Tissue Collection: RNA Stabilization Solution (Ambion) was frozen or used fresh, for the analysis of *β-amy1* activity. Two parallel RNA extractions from three independent biological replicates were carried out according to Li and Trick [24] with the following modifications: the volume of each solution added was increased by 50%, and the acid-phenol-chloroform and chloroform-isoamylacohol extractions were repeated. The RNA pellets were resuspended in 20 µl water containing 1 µl of RNasin® Plus RNase Inhibitor and incubated at 55–60°C for 10 min. For cDNA synthesis, the TaqMan® Reverse Transcription kit (Applied Biosystems, Foster City, USA) was used, primed by random hexamer according to manufacturer’s instructions.

### RT-qPCR

The eight RGs (RG1–RG8) suggested by Faccioli et al. [25] were adopted using and the primer sequences are presented in Table 1. The primer sequences for additional two RGs (RG9 and RG10) were designed using Primer 3 v.0.4.0 from gene sequences available in the EMBL database (Table 1). Primers for *β-amy1* were designed to span an intron in order to detect any contaminating genomic DNA in the cDNA template (TGATAACCAGCCTCTCTTCCA/GACGATAACACCAGCATCCA). Accumulating amplicons were detected by SYBR Green dye staining. Each 25 µl reaction contained 1µl cDNA, 1x Power SYBR Green PCR Master Mix (Applied Biosystems), and 200 nM each primer. The reactions were held at 95°C for 10 minutes and then cycled 40 times at 95°C for 15 s and 62°C for 60 s. The specificity of amplification reactions was verified by melting curve analysis. Serially diluted cDNAs identical to those amplified in the RT-qPCR were prepared to establish reaction efficiency. Expression levels were modeled by the number of amplification cycles required to reach the threshold established after the exponential phase of PCR [26]. The efficiency of the reaction was given by ((10[-1/slope])-1) ×100.

**Table 1:** The primer sequences adopted for RT-qPCR.

Primer	Sequence
RG1	TCGGCTACAGCATTGAAGACG/CCAAAAACGATATCAGGATGGC
RG2	ATGATTCCCACCAAGCCCAT/ACACCAACAGCCACAGTTTGC
RG3	GCCAGTTACTGTCTTTGGCGTC/GGCCTTGTCCTTGTCAGTGAAG
RG4	CGCCCAGTTATCCATCCATCTA/AAAAACACCACAGGACCGGAC
RG5	GCTCAACATGGACCTCTTCAGG/CCGACAAGGACAACATCATGG
RG6	CCCTGTGGAGGCACTACTTTCA/TCACGCAGCTCATCCTCATTC
RG7	TTTGCAGCCCTCGAATCTACC/GCCAATGTAGGCAGCGTTCTT
RG8	CAAGAAGCTTGTCTCTGCCACC/ACAGCCCCTCGAACTTCTCCTT
RG9	AGACCATCACGCTGGAGGTG/GTCGGCGTTGGGGCACTCCTT
RG10	CAGTTGAAGATGCGGCCAC/CAATCATTCCGTACGACCTCC

Two methods were compared to identify suitable RGs: the first was based on a pairwise variation analysis for each RG with geometric averaging as outlined by Vandesompele et al. [22], and the second was a model-based approach as suggested by Anderson et al. [27]. For the former, Ct values were transformed as described by Vandesompele et al. [22], and the results were analyzed by geNorm software (http://alserv.ugent.be/-jvdesomp.genorm/index.html). This allowed for the estimation of an expression stability measure (M). Further pairwise variation Vn/n+1 analysis was carried out to establish the optimal number of RGs required. Alternatively, NormFinder (http://www.multid.se/genex/web_manual/hs410.html) was used to derive a stability value for the identification of optimal RG(s). Finally, RefFinder that compare results and weights of different approaches were employed [28] (http://www.leonxie.com/referencegene.php).

### In Silico Analysis

The UniGene database (http://www.ncbi.nlm.nih.gov/unigene status August 2010) was used to find the frequency of occurrence of different gene transcripts in various barley tissues. UniGene tools (EST profiling) and STATISTICS software were used to analyze the data.

### Enzyme Activity Analysis

*β-amy1* activity of crude protein extracts of the developing caryopsis was determined using the Megazyme Betamyl method [29]. Preparations made in the absence of supplementary cysteine are referred to as “soluble” *β-amy1*, while those containing cysteine supplement are “total” β-*amy1*. Enzyme activity (measured in Betamyl units–U/g of grinded caryopsis) was measured spectrophotometrically at 410 nm, following the kit manufacturer’s instructions (http://www.megazyme.com/booklets/RBAMR6.pdf).

## Results and Discussion

### In Silico Analysis

Candidate RGs selected according to their performance (stability values, overall expression degrees) previously reported by Facciolli et al. [25] and two RGs (ubiquitin and acyl carrier protein III) commonly used in different plant species were firstly analyzed in comparison to UniGene databases (http://www.ncbi.nlm.nih.gov/unigene) build #56 (Apr-2010). All sequences corresponding to each of the candidate RGs were represented among the 23,542 entries present in UniGene database. Following this, the largest clusters that were related to photosynthesis and sugar metabolism and those that were expressed only in some tissues (for example, leaves) were omitted because they cause certain bias. Eight of the ten candidate sequences (except for RG2– elongation factor –1 alpha and RG10– acyl carrier protein III) belonged to the top 100 largest entries in the UniGene database. Each entry is a set of transcript sequences that appears to come from the same transcription locus; therefore their abundance is a good indication that candidate RG belongs to the group of widely expressed genes in various tissues tested under different conditions (Table 2). For this reason they may be considered as appropriate reference gene candidates. Another database was successfully used by Paolacci et al. [7] in wheat.

**Table 2:** Description of the putative reference gene (RGs), their TC numbers and abundances.

RGs	Putative Function ofGene Product	TC Release10.0	Number ofSequences	UniGene	Spike	Pericarp	Pistil	Seed	Average TPM	CV
**RG1**	S-adenosylmethioninedecarboxylase	TC158262	976	Hv.22842	2269	1411	863	1615	1540	0.377
**RG2**	elongation factor 1-alpha	TC176822	119	Hv.26096	421	245	39	429	284	0.648
**RG3**	Glyceraldehyde 3-phosphatedehydrogenase	TC161681	887	Hv.22848	1945	2578	1452	2575	2138	0.255
**RG4**	Glycine rich protein,RNA binding protein	TC163369	367	Hv.21398	1102	368	1295	1276	1010	0.432
**RG5**	Heat shock 70 KD protein(HSP70)	TC159018	837	Hv.19033	2107	1780	1216	1750	1713	0.215
**RG6**	ADP-ribosylation factor	TC177402	390	Hv.22835	1231	982	2041	835	1272	0.423
**RG7**	fructose-bisphosphatealdolase	BY839322	508	Hv.22920	1264	1780	1491	1457	1498	0.142
**RG8**	cytosolic heat shockprotein 90	TC180189	651	Hv.22798	1945	2148	1844	1276	1803	0.207
**RG9**	ubiquitin gene (*mub1*)	TC158625	366	Hv.22903	1556	1903	2473	1129	1765	0.322
**RG10**	acyl carrier protein III	TC170191	127	Hv.65	421	368	824	417	508	0.418

For *in silico* expression, profiles of each RG are available on the UniGene EST ProfileViewer across selected tissues related to barley caryopsis. Average TPM  =  average through all four tissues, CV  =  coefficient of variation of transcript occurrence for the four tissues.

### Reproducibility and RT-qPCR Efficiency

The basic parameter values considered important for a PCR assay described in “Minimum Information for Publication of RT-qPCR Experiments” [4] were evaluated.

We anticipated that a high quality RNA is the basic prerequisite for successful evaluation of gene expression. A modified protocol [24] worked the best compared to other protocols tested, including a TriReagent kit (MRC), TRIzol Reagent (Sigma), and RNeasy Plant Mini Kit (Qiagen). Our protocol allowed acquisition of comparable RNA quality from different developmental stages of barley caryopsis in terms of concentration, purity and integrity (c = 200±35 ng/µl, A_260/280_ = 1.8–2.0 A_260/320_ = 1.9–2.0). Such RNA is appropriate for downstream processing. The importance of the quality parameters of RNA is highlighted by Becker et al [30].

Sources of variation in RT-qPCR background were quantified by estimating the standard deviations of replicates, as recommended by ISO 23025∶2004 and suggested by Huggett et al. [31]. Till date, no report has been published describing effect of individual steps of the reaction, e.g. reverse transcription efficiency, on the final results. We showed that the identified values of reproducibility fit within acceptable criteria values for analytical assay outlined for gene quantification [32]. The reproducibility measure suggested that much of the background variation is linked to the developmental stage reached by individual caryopses (Table 3). Other parameters did not contribute significantly to the overall variability of the assay. It is likely that the developmental stage of each embryo varies to some extent in the first several days depending on their position on the spike and contribution from the environmental factors. Similar findings were described by Sreenivasulu et al. [33] using Affymetrix chips. The RT-qPCR efficiencies represent another important factor. The PCR efficiency has a major impact on the fluorescence history related to Ct and the accuracy of the calculated expression result; it is critically influenced by reaction components for PCR [34]. In our investigation, for individual amplicons, they resulted as follows: RG1–92.0%, RG2–86.6%, RG3–88.8%, RG4–96.3%, RG5–88.3%, RG6–88.5%, RG7–88.7%, RG8–86.3%, RG9–79.6%, RG10–80.6%. This gives a range of 79–96%. Although it is recommended to use reactions with similar efficiencies, a subsequent normalization step to some extent allows the use of amplicons with different efficiencies. SYBRGreen (SG) is used in real-time PCR applications as an intercalating dye and is included in many commercially available kits. It was shown that due to the nature of SYBRGreen, efficiencies in different reactions are affected. Efficiency may be improved by the use of TaqMan or equivalent probes instead of the inexpensive SYBRGreen [35]. The binding of SG to double-stranded DNA is non-specific and additional testing using melting curve analysis needs to be performed to confirm specificity of the reaction. Such analysis proved that only specific products were amplified by designed primer pairs.

**Table 3:** Background variation during the experimental process as assessed by calculating the standard deviation of repetitions across RGs and developmental stages.

SD	5 DPA	10 DPA	15 DPA	20 DPA	25 DPA
plant material	2.606	1.967	1.363	1.349	1.243
parallel RNA extraction	0.337	0.448	0.383	0.206	0.313
qPCR	0.238	0.234	0.234	0.262	0.240
triplicate	0.127	0.113	0.077	0.078	0.104

SD: standard deviation, DPA: number of days after anthesis when the caryopsis was harvested.

The reaction was not inhibited by template quantity over the range of cDNA concentrations tested for nine of the candidate RGs, with the exception of RG1 amplification, which was not found fully linear repeatedly across the range of template concentration tested.

Except for RG1, the assays fulfilled expected performance criteria.

### Gene Expression Stability Analysis

Apart from the quality of the template RNA and the availability of a reproducible PCR assay, RT-qPCR analysis also relies heavily on appropriate RG(s) for normalization [36]. A number of studies have explored the utility of commonly used RGs, and these have shown that the expression of some RGs is too variable for normalization purposes [12], [37], [38]. For this reason, we set out to identify a panel of RGs which could be used for gene expression analysis in the developing barley caryopsis.

**Figure 1:**
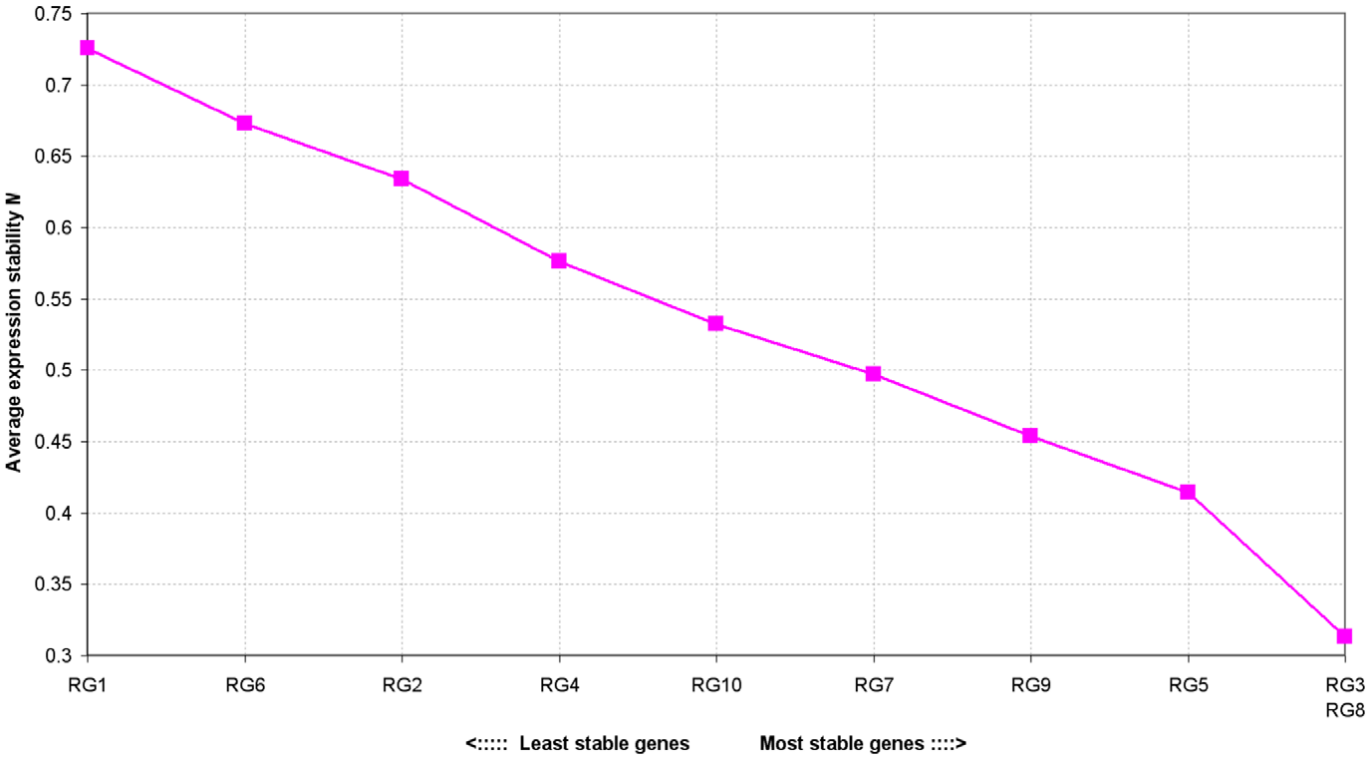
GeNorm analysis of average expression stability values clearly indicate that RG 8 and RG3 are the most stably expressed values when developing barley caryopsis is considered.

**Figure 2:**
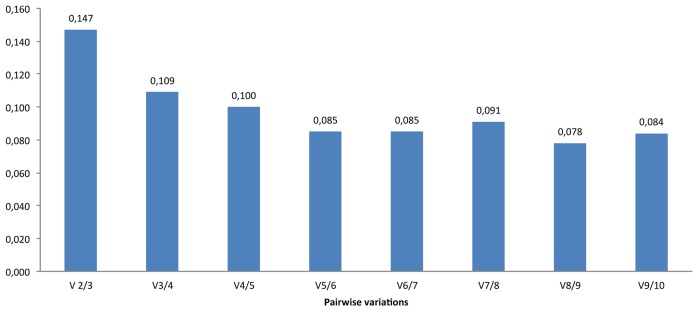
GeNorm analysis showing that 4 genes are optimal for normalization pourposes of gene expression in developing barley caryopsis.

The range of Ct values obtained by RGs varied from 16.39 (RG9) to 24.43 (RG10) on the higher scale, with RG3 being the least variable across all the stages (a difference of 1.90), followed by RG8 (2.01), RG9 (4.84) and RG7 (4.86) were the most variable RGs. A difference of Ct values ≤2 is considered appropriate for normalization [25]. However, calculated stability values (M) started from 0.313, with the best-performing ones being RG3 (M = 0.313) and RG8 (M = 0.313) followed by RG5 (M = 0.464) and RG10 (M = 0.493) while the others showed M>0.500. Such individual stability values (M) are not sufficient for correct normalization (Figs. 1, 2 and Data S1).

**Figure 3:**
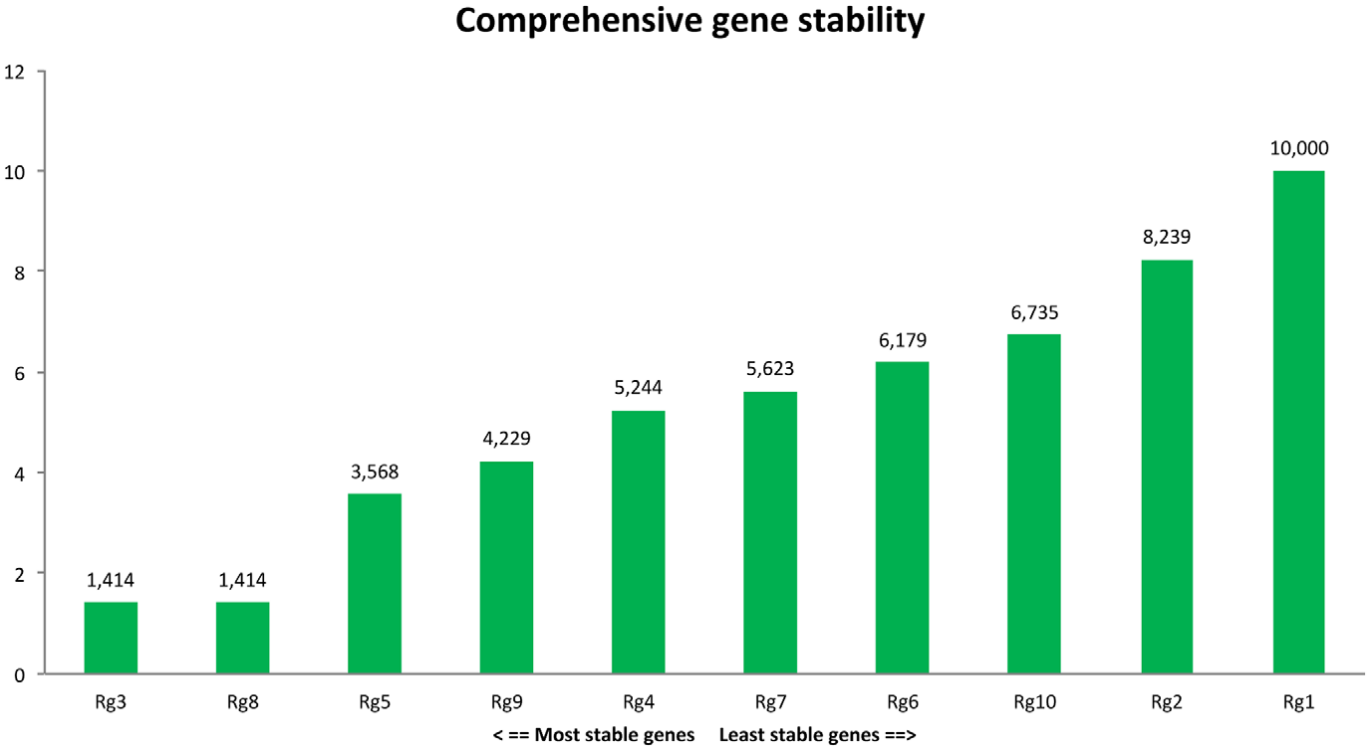
RefFinder based on the rankings from Delta CT, BestKeeper, Normfinder, Genorm each program, It assigns an appropriate weight to an individual gene and calculated the geometric mean of their weights for the overall final ranking.

The best performing single RG (RG3) in our experiments encodes GAPDH, a glycolytic enzyme [39]. Although it was clear that no single RG was sufficient for normalization purposes further supporting the conclusion reached previously other authors [20], [40]. On the other hand the enzyme was identified as being stably expressed by Jarosova and Kundu [14] in barley and was used it to quantify virus infection or by Christensen and Scheller [41] who recommended it as a single gene for normalisation. However, our results clearly showed that a single gene is insufficient for normalization when early stages of development are considered. This enzyme was also identified as being stably expressed in other organisms [42]. HSP90, the second most stable of the RG (RG8) candidates, is a molecular chaperone present in both eukaryotic and prokaryotic cells, and acts as a regulator of signal transduction in the cell cycle [43], [44]. It can represent as much as 1–2% of total cellular protein [2]. The third gene, Hsp70 (encoded by RG5), has remained relatively constant throughout caryopsis development [45]. Faccioli et al. [25] also reported small variations of Ct under stress condition for these two genes across different tissues; thus, our finding is consistent with facts identified by other authors. The final gene among those most stable, ubiquitin, has been widely used as an RG in *A. thaliana* [12] and other plants. UBQ 11 gene expression was shown to be stable during seed imbibition [46] and is used as a RG on the Affymetrix *A. thaliana* gene chip (www.affymetrix.com), although it is generally known that ubiquitin expression may elevate upon certain stimuli [47]. In our study, however, it was inadequate as a RG on its own but can still be included in multi-gene RG set.

Since there was no individual gene that had an M value of <0.15 (the suggested cut-off value for pairwise comparisons), the calculation showed that multiple RGs-optimally four-would be necessary for effective normalization. The use of four RGs reduced the M value to <0.1 (Fig. 2).

The model-based strategy ranked the RGs according to their expression stability and it also identified RG3 and RG5 as the best-performing genes (Data S2) followed by RG8. The pair RG3 and RG5 was, according to model based strategy, the most suited for normalization (stability value of 0.10 according to the algorithm).

Principal differences were not observed in the GeNorm and NormFinder evaluation of the best sets of RGs for each developmental stage tested. Both non-normalized measurements and the model-based approaches selected the set RG3, RG5, RG8 and RG9, although their order differed slightly. In addition, other authors [48], [49] reported certain inconsistencies between the two methods because they had used different statistical algorithms. To prove the results, RefFinder software was employed that used to assign appropriate weight to an individual gene based on the ranking of frequently used programs including geNORM, NormFinder, BestKeeper and the comparative delta-Ct method (Fig. 3) This approach confirmed our finding and can be recommended for the selection of best RGs. Other candidate RGs were proved not to be suitable for normalization of gene expression in developing caryopses, although for other plant and tissue samples, they worked as expected [50]. Elongation factor 1 alpha that is used as a RG for normalization of Affymetrix arrays [51] was less stable according to both algorithms used. We again emphasize the importance of identifying the most stable RGs for particular tissues, an issue suggested previously as well [52], [53].

Transcript frequency *in silico* (measured in TPM  =  transcripts per million) and its variability across tissues (measured by its CV  =  coefficient of variation) of the candidate RGs are fully consistent with the ordering of candidate RGs provided by the model-based approach. Thus, the RG3 and RG8 sequences have shown a low TPM, CV and selected TPM sum. The stability values of the various RGs were significantly (p<0.05) correlated with their TPM values within pistil (r = −0.663), pericarp (r = −0.843), stem + spike + pistil + pericarp + caryopsis (r = −0.732), and spike + pistil + pericarp + caryopsis (r = −0.784) libraries, with a CV of 0.21 of this set of resources. Thus, *in silico* evaluation may help to predict and select RG for particular tissue (Table 2).

### β-amy1 Expression

To establish an assay for *β-amy1* gene expression studies and demonstrate the usability of the RG panel, we tracked expression of the *β-amy1* gene. The gene is transcribed during caryopsis development and impacts the final malting quality of barley grains. A qPCR assay was optimized stepwise and when used as it is described in the material and methods section showed the results were reproducible and robust (efficiency 96%, reproducibility 95%, calibration curve slope –3.23 regardless of which cultivar was used). Specificity can be shown by melting curve analysis. The gene was not transcribed into mRNA 5 DAP, but a detectable amount was found 10 DAP, with significant increase in subsequent days (15, 20 and 25 DAP). No protein activity was detectable 5 DAP by enzymatic assay, but its presence became detectable by 10 DAP and since has increased as it as enzymatic activity measurement shows (Tab. 3). Both assays are fully consistent to each other and the protein assay validates transcription assay results. A high expression of *β-amy1* 17, 19 and 21st day after anthesis was very recently reported by Vinje et al. [54]. They investigated four cultivars differing in the degree of expression; however, they included genotypes with different alleles and genetic backgrounds [55], whereas for assay validation, we selected three spring malting barley cultivars with similar pedigree and identical allele [23]. Thus, no difference was observed. The work by Vinje and co-authors also supports our findings that RG8 (cytosolic HSP90) is one of the RGs suited for the normalization of gene expression in developing barley grains from early stages up to the maturity.

Considering the fact that ΒMY1 is important for barley grain quality, the assay can be used to study expression of *β-amy1* alleles with different thermostabilities under different environmental conditions which can provide crucial information for barley breeders and breweries.

## Supporting Information

Data S1 Three individual plants per data point were used, from each plant two independent RNA isolations were performed. Ct values were calculated as mean of three different measurement (triplicate analysis).
(DOC)

Data S2 Reported values are the mean of three measures.
(DOC)
