# Supplementary material for: Validation of the β-amy1 Transcription Profiling Assay and Selection of Reference Genes Suited for a RT-qPCR Assay in Developing Barley Caryopsis
Source: PLoS One. 2012 Jul 31;7(7):e41886. doi: 10.1371/journal.pone.0041886 (PMC3409215; doi:10.1371/journal.pone.0041886)

|  | RG1 | RG2 | RG3 | RG4 | RG5 | RG6 | RG7 | RG8 | RG9 | RG10 |
| --- | --- | --- | --- | --- | --- | --- | --- | --- | --- | --- |
| 5 DPA,RNA 1 | 1 | 0.193988056 | 0.638402278 | 0.587648597 | 0.394109744 | 0.423425066 | 0.581064375 | 0.744699996 | 0.640827394 | 0.649194316 |
| 5 DPA,RNA 2 | 0.390882503 | 0.113948766 | 0.618402644 | 0.564393937 | 0.425124481 | 0.182745623 | 0.667634816 | 0.423374379 | 0.7611458 | 0.573809168 |
| 5 DPA,RNA 3 | 0.585723963 | 0.310210287 | 0.678200803 | 0.658872279 | 0.457134782 | 0.462893643 | 0.605402918 | 0.479321777 | 0.898706446 | 1 |
| 10 DPA,RNA 1 | 0.239646069 | 1 | 1 | 0.350010061 | 1 | 1 | 0.531914894 | 1 | 0.928517681 | 0.398564423 |
| 10 DPA,RNA 2 | 0.524241966 | 0.30731133 | 0.665371884 | 0.71188638 | 0.464406418 | 0.445546985 | 0.655110018 | 0.516379169 | 0.931276343 | 0.850746285 |
| 10 DPA,RNA 3 | 0.451203543 | 0.128741551 | 0.620374084 | 0.585674645 | 0.429169136 | 0.227628326 | 1 | 0.473409427 | 1 | 0.711118451 |
| 15 DPA,RNA 1 | 0.185210807 | 0.288665187 | 0.750917435 | 0.566296164 | 0.310053894 | 0.411467732 | 0.319996392 | 0.54604053 | 0.420522542 | 0.528480944 |
| 15 DPA,RNA 2 | 0.125223427 | 0.36162412 | 0.604776872 | 0.601653803 | 0.536128783 | 0.750917435 | 0.288342751 | 0.643644125 | 0.772520157 | 0.448283785 |
| 15 DPA,RNA 3 | 0.13022179 | 0.094736285 | 0.406262323 | 0.501692406 | 0.158792457 | 0.428850381 | 0.212967469 | 0.27590568 | 0.38243771 | 0.203334706 |
| 20 DPA,RNA 1 | 0.115042001 | 0.06568845 | 0.406262323 | 0.182832509 | 0.121426306 | 0.230544912 | 0.1318115 | 0.214589219 | 0.135803698 | 0.221424679 |
| 20 DPA,RNA 2 | 0.109192379 | 0.125558198 | 0.370441459 | 0.313226341 | 0.169139738 | 0.350929867 | 0.180730824 | 0.349281481 | 0.321983726 | 0.239007456 |
| 20 DPA,RNA 3 | 0.065647376 | 0.086516575 | 0.277286972 | 0.199547692 | 0.088839202 | 0.249639479 | 0.100794357 | 0.180922191 | 0.203297963 | 0.17919584 |
| 25 DPA,RNA 1 | 0.424090832 | 0.254698325 | 0.524072376 | 0.966912548 | 0.294784444 | 0.490188117 | 0.314985916 | 0.358060223 | 0.497998087 | 0.178669968 |
| 25 DPA,RNA 2 | 0.083024413 | 0.06863062 | 0.336705581 | 0.335030144 | 0.133908224 | 0.371622409 | 0.153373803 | 0.260918261 | 0.277596687 | 0.201549824 |
| 25 DPA,RNA 3 | 0.209649368 | 0.320072437 | 0.920576672 | 1 | 0.399117094 | 0.758122027 | 0.426468446 | 0.566755386 | 0.662084454 | 0.510167727 |

| 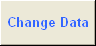   | 1.5 | | --- | | RG1 | RG2 | RG3 | RG4 | RG5 | RG6 | RG7 | RG8 | RG9 | RG10 | **Normalisation Factor** |
| --- | --- | --- | --- | --- | --- | --- | --- | --- | --- | --- | --- | --- |
| 5 DPA,RNA 1 | 1.00E+00 | 1.94E-01 | 6.38E-01 | 5.88E-01 | 3.94E-01 | 4.23E-01 | 5.81E-01 | 7.45E-01 | 6.41E-01 | 6.49E-01 | **1.4969** |
| 5 DPA,RNA 2 | 3.91E-01 | 1.14E-01 | 6.18E-01 | 5.64E-01 | 4.25E-01 | 1.83E-01 | 6.68E-01 | 4.23E-01 | 7.61E-01 | 5.74E-01 | **1.1444** |
| 5 DPA,RNA 3 | 5.86E-01 | 3.10E-01 | 6.78E-01 | 6.59E-01 | 4.57E-01 | 4.63E-01 | 6.05E-01 | 4.79E-01 | 8.99E-01 | 1.00E+00 | **1.6083** |
| 10 DPA,RNA 1 | 2.40E-01 | 1.00E+00 | 1.00E+00 | 3.50E-01 | 1.00E+00 | 1.00E+00 | 5.32E-01 | 1.00E+00 | 9.29E-01 | 3.99E-01 | **1.8323** |
| 10 DPA,RNA 2 | 5.24E-01 | 3.07E-01 | 6.65E-01 | 7.12E-01 | 4.64E-01 | 4.46E-01 | 6.55E-01 | 5.16E-01 | 9.31E-01 | 8.51E-01 | **1.5991** |
| 10 DPA,RNA 3 | 4.51E-01 | 1.29E-01 | 6.20E-01 | 5.86E-01 | 4.29E-01 | 2.28E-01 | 1.00E+00 | 4.73E-01 | 1.00E+00 | 7.11E-01 | **1.3346** |
| 15 DPA,RNA 1 | 1.85E-01 | 2.89E-01 | 7.51E-01 | 5.66E-01 | 3.10E-01 | 4.11E-01 | 3.20E-01 | 5.46E-01 | 4.21E-01 | 5.28E-01 | **1.1127** |
| 15 DPA,RNA 2 | 1.25E-01 | 3.62E-01 | 6.05E-01 | 6.02E-01 | 5.36E-01 | 7.51E-01 | 2.88E-01 | 6.44E-01 | 7.73E-01 | 4.48E-01 | **1.2712** |
| 15 DPA,RNA 3 | 1.30E-01 | 9.47E-02 | 4.06E-01 | 5.02E-01 | 1.59E-01 | 4.29E-01 | 2.13E-01 | 2.76E-01 | 3.82E-01 | 2.03E-01 | **0.6770** |
| 20 DPA,RNA 1 | 1.15E-01 | 6.57E-02 | 4.06E-01 | 1.83E-01 | 1.21E-01 | 2.31E-01 | 1.32E-01 | 2.15E-01 | 1.36E-01 | 2.21E-01 | **0.4506** |
| 20 DPA,RNA 2 | 1.09E-01 | 1.26E-01 | 3.70E-01 | 3.13E-01 | 1.69E-01 | 3.51E-01 | 1.81E-01 | 3.49E-01 | 3.22E-01 | 2.39E-01 | **0.6418** |
| 20 DPA,RNA 3 | 6.56E-02 | 8.65E-02 | 2.77E-01 | 2.00E-01 | 8.88E-02 | 2.50E-01 | 1.01E-01 | 1.81E-01 | 2.03E-01 | 1.79E-01 | **0.4053** |
| 25 DPA,RNA 1 | 4.24E-01 | 2.55E-01 | 5.24E-01 | 9.67E-01 | 2.95E-01 | 4.90E-01 | 3.15E-01 | 3.58E-01 | 4.98E-01 | 1.79E-01 | **1.0745** |
| 25 DPA,RNA 2 | 8.30E-02 | 6.86E-02 | 3.37E-01 | 3.35E-01 | 1.34E-01 | 3.72E-01 | 1.53E-01 | 2.61E-01 | 2.78E-01 | 2.02E-01 | **0.5330** |
| 25 DPA,RNA 3 | 2.10E-01 | 3.20E-01 | 9.21E-01 | 1.00E+00 | 3.99E-01 | 7.58E-01 | 4.26E-01 | 5.67E-01 | 6.62E-01 | 5.10E-01 | **1.4440** |
|  | 0.228302982 | 0.18563312 | 0.552975913 | 0.486400203 | 0.295747474 | 0.406003788 | 0.336288616 | 0.423987874 | 0.507644029 | 0.390678278 |  |
| **M < 1.5** | **0.936** | **0.839** | **0.610** | **0.750** | **0.622** | **0.865** | **0.688** | **0.592** | **0.613** | **0.739** |  |

| 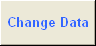   | 1.5 | | --- | | RG2 | RG3 | RG4 | RG5 | RG6 | RG7 | RG8 | RG9 | RG10 | **Normalisation Factor** |
| --- | --- | --- | --- | --- | --- | --- | --- | --- | --- | --- | --- |
| 5 DPA,RNA 1 | 1.94E-01 | 6.38E-01 | 5.88E-01 | 3.94E-01 | 4.23E-01 | 5.81E-01 | 7.45E-01 | 6.41E-01 | 6.49E-01 | **1.3286** |
| 5 DPA,RNA 2 | 1.14E-01 | 6.18E-01 | 5.64E-01 | 4.25E-01 | 1.83E-01 | 6.68E-01 | 4.23E-01 | 7.61E-01 | 5.74E-01 | **1.0943** |
| 5 DPA,RNA 3 | 3.10E-01 | 6.78E-01 | 6.59E-01 | 4.57E-01 | 4.63E-01 | 6.05E-01 | 4.79E-01 | 8.99E-01 | 1.00E+00 | **1.5269** |
| 10 DPA,RNA 1 | 1.00E+00 | 1.00E+00 | 3.50E-01 | 1.00E+00 | 1.00E+00 | 5.32E-01 | 1.00E+00 | 9.29E-01 | 3.99E-01 | **1.9493** |
| 10 DPA,RNA 2 | 3.07E-01 | 6.65E-01 | 7.12E-01 | 4.64E-01 | 4.46E-01 | 6.55E-01 | 5.16E-01 | 9.31E-01 | 8.51E-01 | **1.5361** |
| 10 DPA,RNA 3 | 1.29E-01 | 6.20E-01 | 5.86E-01 | 4.29E-01 | 2.28E-01 | 1.00E+00 | 4.73E-01 | 1.00E+00 | 7.11E-01 | **1.2777** |
| 15 DPA,RNA 1 | 2.89E-01 | 7.51E-01 | 5.66E-01 | 3.10E-01 | 4.11E-01 | 3.20E-01 | 5.46E-01 | 4.21E-01 | 5.28E-01 | **1.1525** |
| 15 DPA,RNA 2 | 3.62E-01 | 6.05E-01 | 6.02E-01 | 5.36E-01 | 7.51E-01 | 2.88E-01 | 6.44E-01 | 7.73E-01 | 4.48E-01 | **1.3956** |
| 15 DPA,RNA 3 | 9.47E-02 | 4.06E-01 | 5.02E-01 | 1.59E-01 | 4.29E-01 | 2.13E-01 | 2.76E-01 | 3.82E-01 | 2.03E-01 | **0.6900** |
| 20 DPA,RNA 1 | 6.57E-02 | 4.06E-01 | 1.83E-01 | 1.21E-01 | 2.31E-01 | 1.32E-01 | 2.15E-01 | 1.36E-01 | 2.21E-01 | **0.4451** |
| 20 DPA,RNA 2 | 1.26E-01 | 3.70E-01 | 3.13E-01 | 1.69E-01 | 3.51E-01 | 1.81E-01 | 3.49E-01 | 3.22E-01 | 2.39E-01 | **0.6631** |
| 20 DPA,RNA 3 | 8.65E-02 | 2.77E-01 | 2.00E-01 | 8.88E-02 | 2.50E-01 | 1.01E-01 | 1.81E-01 | 2.03E-01 | 1.79E-01 | **0.4211** |
| 25 DPA,RNA 1 | 2.55E-01 | 5.24E-01 | 9.67E-01 | 2.95E-01 | 4.90E-01 | 3.15E-01 | 3.58E-01 | 4.98E-01 | 1.79E-01 | **1.0110** |
| 25 DPA,RNA 2 | 6.86E-02 | 3.37E-01 | 3.35E-01 | 1.34E-01 | 3.72E-01 | 1.53E-01 | 2.61E-01 | 2.78E-01 | 2.02E-01 | **0.5561** |
| 25 DPA,RNA 3 | 3.20E-01 | 9.21E-01 | 1.00E+00 | 3.99E-01 | 7.58E-01 | 4.26E-01 | 5.67E-01 | 6.62E-01 | 5.10E-01 | **1.5185** |
|  | 0.18563312 | 0.552975913 | 0.486400203 | 0.295747474 | 0.406003788 | 0.336288616 | 0.423987874 | 0.507644029 | 0.390678278 |  |
| **M < 1.5** | **0.790** | **0.568** | **0.732** | **0.587** | **0.808** | **0.701** | **0.549** | **0.588** | **0.731** |  |

| 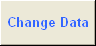   | 1.5 | | --- | | RG2 | RG3 | RG4 | RG5 | RG7 | RG8 | RG9 | RG10 | **Normalisation Factor** |
| --- | --- | --- | --- | --- | --- | --- | --- | --- | --- | --- |
| 5 DPA,RNA 1 | 1.94E-01 | 6.38E-01 | 5.88E-01 | 3.94E-01 | 5.81E-01 | 7.45E-01 | 6.41E-01 | 6.49E-01 | **1.3694** |
| 5 DPA,RNA 2 | 1.14E-01 | 6.18E-01 | 5.64E-01 | 4.25E-01 | 6.68E-01 | 4.23E-01 | 7.61E-01 | 5.74E-01 | **1.2228** |
| 5 DPA,RNA 3 | 3.10E-01 | 6.78E-01 | 6.59E-01 | 4.57E-01 | 6.05E-01 | 4.79E-01 | 8.99E-01 | 1.00E+00 | **1.5837** |
| 10 DPA,RNA 1 | 1.00E+00 | 1.00E+00 | 3.50E-01 | 1.00E+00 | 5.32E-01 | 1.00E+00 | 9.29E-01 | 3.99E-01 | **1.8932** |
| 10 DPA,RNA 2 | 3.07E-01 | 6.65E-01 | 7.12E-01 | 4.64E-01 | 6.55E-01 | 5.16E-01 | 9.31E-01 | 8.51E-01 | **1.6020** |
| 10 DPA,RNA 3 | 1.29E-01 | 6.20E-01 | 5.86E-01 | 4.29E-01 | 1.00E+00 | 4.73E-01 | 1.00E+00 | 7.11E-01 | **1.4162** |
| 15 DPA,RNA 1 | 2.89E-01 | 7.51E-01 | 5.66E-01 | 3.10E-01 | 3.20E-01 | 5.46E-01 | 4.21E-01 | 5.28E-01 | **1.1712** |
| 15 DPA,RNA 2 | 3.62E-01 | 6.05E-01 | 6.02E-01 | 5.36E-01 | 2.88E-01 | 6.44E-01 | 7.73E-01 | 4.48E-01 | **1.3473** |
| 15 DPA,RNA 3 | 9.47E-02 | 4.06E-01 | 5.02E-01 | 1.59E-01 | 2.13E-01 | 2.76E-01 | 3.82E-01 | 2.03E-01 | **0.6542** |
| 20 DPA,RNA 1 | 6.57E-02 | 4.06E-01 | 1.83E-01 | 1.21E-01 | 1.32E-01 | 2.15E-01 | 1.36E-01 | 2.21E-01 | **0.4317** |
| 20 DPA,RNA 2 | 1.26E-01 | 3.70E-01 | 3.13E-01 | 1.69E-01 | 1.81E-01 | 3.49E-01 | 3.22E-01 | 2.39E-01 | **0.6415** |
| 20 DPA,RNA 3 | 8.65E-02 | 2.77E-01 | 2.00E-01 | 8.88E-02 | 1.01E-01 | 1.81E-01 | 2.03E-01 | 1.79E-01 | **0.4016** |
| 25 DPA,RNA 1 | 2.55E-01 | 5.24E-01 | 9.67E-01 | 2.95E-01 | 3.15E-01 | 3.58E-01 | 4.98E-01 | 1.79E-01 | **0.9889** |
| 25 DPA,RNA 2 | 6.86E-02 | 3.37E-01 | 3.35E-01 | 1.34E-01 | 1.53E-01 | 2.61E-01 | 2.78E-01 | 2.02E-01 | **0.5225** |
| 25 DPA,RNA 3 | 3.20E-01 | 9.21E-01 | 1.00E+00 | 3.99E-01 | 4.26E-01 | 5.67E-01 | 6.62E-01 | 5.10E-01 | **1.4798** |
|  | 0.18563312 | 0.552975913 | 0.486400203 | 0.295747474 | 0.336288616 | 0.423987874 | 0.507644029 | 0.390678278 |  |
| **M < 1.5** | **0.808** | **0.565** | **0.726** | **0.554** | **0.642** | **0.544** | **0.548** | **0.686** |  |

| 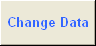   | 1.5 | | --- | | RG3 | RG4 | RG5 | RG7 | RG8 | RG9 | RG10 | **Normalisation Factor** |
| --- | --- | --- | --- | --- | --- | --- | --- | --- | --- |
| 5 DPA,RNA 1 | 6.38E-01 | 5.88E-01 | 3.94E-01 | 5.81E-01 | 7.45E-01 | 6.41E-01 | 6.49E-01 | **1.4233** |
| 5 DPA,RNA 2 | 6.18E-01 | 5.64E-01 | 4.25E-01 | 6.68E-01 | 4.23E-01 | 7.61E-01 | 5.74E-01 | **1.3493** |
| 5 DPA,RNA 3 | 6.78E-01 | 6.59E-01 | 4.57E-01 | 6.05E-01 | 4.79E-01 | 8.99E-01 | 1.00E+00 | **1.5716** |
| 10 DPA,RNA 1 | 1.00E+00 | 3.50E-01 | 1.00E+00 | 5.32E-01 | 1.00E+00 | 9.29E-01 | 3.99E-01 | **1.6304** |
| 10 DPA,RNA 2 | 6.65E-01 | 7.12E-01 | 4.64E-01 | 6.55E-01 | 5.16E-01 | 9.31E-01 | 8.51E-01 | **1.5946** |
| 10 DPA,RNA 3 | 6.20E-01 | 5.86E-01 | 4.29E-01 | 1.00E+00 | 4.73E-01 | 1.00E+00 | 7.11E-01 | **1.5683** |
| 15 DPA,RNA 1 | 7.51E-01 | 5.66E-01 | 3.10E-01 | 3.20E-01 | 5.46E-01 | 4.21E-01 | 5.28E-01 | **1.1247** |
| 15 DPA,RNA 2 | 6.05E-01 | 6.02E-01 | 5.36E-01 | 2.88E-01 | 6.44E-01 | 7.73E-01 | 4.48E-01 | **1.2782** |
| 15 DPA,RNA 3 | 4.06E-01 | 5.02E-01 | 1.59E-01 | 2.13E-01 | 2.76E-01 | 3.82E-01 | 2.03E-01 | **0.6778** |
| 20 DPA,RNA 1 | 4.06E-01 | 1.83E-01 | 1.21E-01 | 1.32E-01 | 2.15E-01 | 1.36E-01 | 2.21E-01 | **0.4442** |
| 20 DPA,RNA 2 | 3.70E-01 | 3.13E-01 | 1.69E-01 | 1.81E-01 | 3.49E-01 | 3.22E-01 | 2.39E-01 | **0.6366** |
| 20 DPA,RNA 3 | 2.77E-01 | 2.00E-01 | 8.88E-02 | 1.01E-01 | 1.81E-01 | 2.03E-01 | 1.79E-01 | **0.3931** |
| 25 DPA,RNA 1 | 5.24E-01 | 9.67E-01 | 2.95E-01 | 3.15E-01 | 3.58E-01 | 4.98E-01 | 1.79E-01 | **0.9437** |
| 25 DPA,RNA 2 | 3.37E-01 | 3.35E-01 | 1.34E-01 | 1.53E-01 | 2.61E-01 | 2.78E-01 | 2.02E-01 | **0.5490** |
| 25 DPA,RNA 3 | 9.21E-01 | 1.00E+00 | 3.99E-01 | 4.26E-01 | 5.67E-01 | 6.62E-01 | 5.10E-01 | **1.4478** |
|  | 0.552975913 | 0.486400203 | 0.295747474 | 0.336288616 | 0.423987874 | 0.507644029 | 0.390678278 |  |
| **M < 1.5** | **0.541** | **0.687** | **0.552** | **0.581** | **0.532** | **0.508** | **0.633** |  |

| 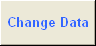   | 1.5 | | --- | | RG3 | RG5 | RG7 | RG8 | RG9 | RG10 | **Normalisation Factor** |
| --- | --- | --- | --- | --- | --- | --- | --- | --- |
| 5 DPA,RNA 1 | 6.38E-01 | 3.94E-01 | 5.81E-01 | 7.45E-01 | 6.41E-01 | 6.49E-01 | **1.4627** |
| 5 DPA,RNA 2 | 6.18E-01 | 4.25E-01 | 6.68E-01 | 4.23E-01 | 7.61E-01 | 5.74E-01 | **1.3837** |
| 5 DPA,RNA 3 | 6.78E-01 | 4.57E-01 | 6.05E-01 | 4.79E-01 | 8.99E-01 | 1.00E+00 | **1.6110** |
| 10 DPA,RNA 1 | 1.00E+00 | 1.00E+00 | 5.32E-01 | 1.00E+00 | 9.29E-01 | 3.99E-01 | **1.8686** |
| 10 DPA,RNA 2 | 6.65E-01 | 4.64E-01 | 6.55E-01 | 5.16E-01 | 9.31E-01 | 8.51E-01 | **1.6175** |
| 10 DPA,RNA 3 | 6.20E-01 | 4.29E-01 | 1.00E+00 | 4.73E-01 | 1.00E+00 | 7.11E-01 | **1.6389** |
| 15 DPA,RNA 1 | 7.51E-01 | 3.10E-01 | 3.20E-01 | 5.46E-01 | 4.21E-01 | 5.28E-01 | **1.1182** |
| 15 DPA,RNA 2 | 6.05E-01 | 5.36E-01 | 2.88E-01 | 6.44E-01 | 7.73E-01 | 4.48E-01 | **1.2852** |
| 15 DPA,RNA 3 | 4.06E-01 | 1.59E-01 | 2.13E-01 | 2.76E-01 | 3.82E-01 | 2.03E-01 | **0.6320** |
| 20 DPA,RNA 1 | 4.06E-01 | 1.21E-01 | 1.32E-01 | 2.15E-01 | 1.36E-01 | 2.21E-01 | **0.4567** |
| 20 DPA,RNA 2 | 3.70E-01 | 1.69E-01 | 1.81E-01 | 3.49E-01 | 3.22E-01 | 2.39E-01 | **0.6354** |
| 20 DPA,RNA 3 | 2.77E-01 | 8.88E-02 | 1.01E-01 | 1.81E-01 | 2.03E-01 | 1.79E-01 | **0.3904** |
| 25 DPA,RNA 1 | 5.24E-01 | 2.95E-01 | 3.15E-01 | 3.58E-01 | 4.98E-01 | 1.79E-01 | **0.8335** |
| 25 DPA,RNA 2 | 3.37E-01 | 1.34E-01 | 1.53E-01 | 2.61E-01 | 2.78E-01 | 2.02E-01 | **0.5287** |
| 25 DPA,RNA 3 | 9.21E-01 | 3.99E-01 | 4.26E-01 | 5.67E-01 | 6.62E-01 | 5.10E-01 | **1.3656** |
|  | 0.552975913 | 0.295747474 | 0.336288616 | 0.423987874 | 0.507644029 | 0.390678278 |  |
| **M < 1.5** | **0.536** | **0.507** | **0.552** | **0.506** | **0.489** | **0.602** |  |

| 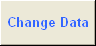   | 1.5 | | --- | | RG3 | RG5 | RG7 | RG8 | RG9 | **Normalisation Factor** |
| --- | --- | --- | --- | --- | --- | --- | --- |
| 5 DPA,RNA 1 | 6.38E-01 | 3.94E-01 | 5.81E-01 | 7.45E-01 | 6.41E-01 | **1.4259** |
| 5 DPA,RNA 2 | 6.18E-01 | 4.25E-01 | 6.68E-01 | 4.23E-01 | 7.61E-01 | **1.3673** |
| 5 DPA,RNA 3 | 6.78E-01 | 4.57E-01 | 6.05E-01 | 4.79E-01 | 8.99E-01 | **1.4685** |
| 10 DPA,RNA 1 | 1.00E+00 | 1.00E+00 | 5.32E-01 | 1.00E+00 | 9.29E-01 | **2.1090** |
| 10 DPA,RNA 2 | 6.65E-01 | 4.64E-01 | 6.55E-01 | 5.16E-01 | 9.31E-01 | **1.5241** |
| 10 DPA,RNA 3 | 6.20E-01 | 4.29E-01 | 1.00E+00 | 4.73E-01 | 1.00E+00 | **1.6049** |
| 15 DPA,RNA 1 | 7.51E-01 | 3.10E-01 | 3.20E-01 | 5.46E-01 | 4.21E-01 | **1.0764** |
| 15 DPA,RNA 2 | 6.05E-01 | 5.36E-01 | 2.88E-01 | 6.44E-01 | 7.73E-01 | **1.3147** |
| 15 DPA,RNA 3 | 4.06E-01 | 1.59E-01 | 2.13E-01 | 2.76E-01 | 3.82E-01 | **0.6571** |
| 20 DPA,RNA 1 | 4.06E-01 | 1.21E-01 | 1.32E-01 | 2.15E-01 | 1.36E-01 | **0.4374** |
| 20 DPA,RNA 2 | 3.70E-01 | 1.69E-01 | 1.81E-01 | 3.49E-01 | 3.22E-01 | **0.6403** |
| 20 DPA,RNA 3 | 2.77E-01 | 8.88E-02 | 1.01E-01 | 1.81E-01 | 2.03E-01 | **0.3780** |
| 25 DPA,RNA 1 | 5.24E-01 | 2.95E-01 | 3.15E-01 | 3.58E-01 | 4.98E-01 | **0.9398** |
| 25 DPA,RNA 2 | 3.37E-01 | 1.34E-01 | 1.53E-01 | 2.61E-01 | 2.78E-01 | **0.5312** |
| 25 DPA,RNA 3 | 9.21E-01 | 3.99E-01 | 4.26E-01 | 5.67E-01 | 6.62E-01 | **1.3779** |
|  | 0.552975913 | 0.295747474 | 0.336288616 | 0.423987874 | 0.507644029 |  |
| **M < 1.5** | **0.518** | **0.465** | **0.562** | **0.472** | **0.468** |  |

| 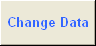   | 1.5 | | --- | | RG3 | RG5 | RG8 | RG9 | **Normalisation Factor** |
| --- | --- | --- | --- | --- | --- | --- |
| 5 DPA,RNA 1 | 6.38E-01 | 3.94E-01 | 7.45E-01 | 6.41E-01 | **1.3590** |
| 5 DPA,RNA 2 | 6.18E-01 | 4.25E-01 | 4.23E-01 | 7.61E-01 | **1.2455** |
| 5 DPA,RNA 3 | 6.78E-01 | 4.57E-01 | 4.79E-01 | 8.99E-01 | **1.3957** |
| 10 DPA,RNA 1 | 1.00E+00 | 1.00E+00 | 1.00E+00 | 9.29E-01 | **2.2663** |
| 10 DPA,RNA 2 | 6.65E-01 | 4.64E-01 | 5.16E-01 | 9.31E-01 | **1.4334** |
| 10 DPA,RNA 3 | 6.20E-01 | 4.29E-01 | 4.73E-01 | 1.00E+00 | **1.3756** |
| 15 DPA,RNA 1 | 7.51E-01 | 3.10E-01 | 5.46E-01 | 4.21E-01 | **1.1101** |
| 15 DPA,RNA 2 | 6.05E-01 | 5.36E-01 | 6.44E-01 | 7.73E-01 | **1.4629** |
| 15 DPA,RNA 3 | 4.06E-01 | 1.59E-01 | 2.76E-01 | 3.82E-01 | **0.6631** |
| 20 DPA,RNA 1 | 4.06E-01 | 1.21E-01 | 2.15E-01 | 1.36E-01 | **0.4495** |
| 20 DPA,RNA 2 | 3.70E-01 | 1.69E-01 | 3.49E-01 | 3.22E-01 | **0.6689** |
| 20 DPA,RNA 3 | 2.77E-01 | 8.88E-02 | 1.81E-01 | 2.03E-01 | **0.4005** |
| 25 DPA,RNA 1 | 5.24E-01 | 2.95E-01 | 3.58E-01 | 4.98E-01 | **0.9406** |
| 25 DPA,RNA 2 | 3.37E-01 | 1.34E-01 | 2.61E-01 | 2.78E-01 | **0.5519** |
| 25 DPA,RNA 3 | 9.21E-01 | 3.99E-01 | 5.67E-01 | 6.62E-01 | **1.4068** |
|  | 0.552975913 | 0.295747474 | 0.423987874 | 0.507644029 |  |
| **M < 1.5** | **0.471** | **0.440** | **0.410** | **0.493** |  |

| 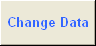   | 1.5 | | --- | | RG3 | RG5 | RG8 | **Normalisation Factor** |
| --- | --- | --- | --- | --- | --- |
| 5 DPA,RNA 1 | 6.38E-01 | 3.94E-01 | 7.45E-01 | **1.3928** |
| 5 DPA,RNA 2 | 6.18E-01 | 4.25E-01 | 4.23E-01 | **1.1709** |
| 5 DPA,RNA 3 | 6.78E-01 | 4.57E-01 | 4.79E-01 | **1.2893** |
| 10 DPA,RNA 1 | 1.00E+00 | 1.00E+00 | 1.00E+00 | **2.4341** |
| 10 DPA,RNA 2 | 6.65E-01 | 4.64E-01 | 5.16E-01 | **1.3202** |
| 10 DPA,RNA 3 | 6.20E-01 | 4.29E-01 | 4.73E-01 | **1.2204** |
| 15 DPA,RNA 1 | 7.51E-01 | 3.10E-01 | 5.46E-01 | **1.2239** |
| 15 DPA,RNA 2 | 6.05E-01 | 5.36E-01 | 6.44E-01 | **1.4438** |
| 15 DPA,RNA 3 | 4.06E-01 | 1.59E-01 | 2.76E-01 | **0.6355** |
| 20 DPA,RNA 1 | 4.06E-01 | 1.21E-01 | 2.15E-01 | **0.5345** |
| 20 DPA,RNA 2 | 3.70E-01 | 1.69E-01 | 3.49E-01 | **0.6809** |
| 20 DPA,RNA 3 | 2.77E-01 | 8.88E-02 | 1.81E-01 | **0.4006** |
| 25 DPA,RNA 1 | 5.24E-01 | 2.95E-01 | 3.58E-01 | **0.9274** |
| 25 DPA,RNA 2 | 3.37E-01 | 1.34E-01 | 2.61E-01 | **0.5536** |
| 25 DPA,RNA 3 | 9.21E-01 | 3.99E-01 | 5.67E-01 | **1.4428** |
|  | 0.552975913 | 0.295747474 | 0.423987874 |  |
| **M < 1.5** | **0.419** | **0.464** | **0.359** |  |

| 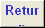   |  | | --- | | **RG1** | **RG2** | **RG3** | **RG4** | **RG5** | **RG6** | **RG7** | **RG8** | **RG9** | **RG10** |
| --- | --- | --- | --- | --- | --- | --- | --- | --- | --- | --- | --- |
| **RG1** |  | 1.230 | 0.943 | 0.889 | 0.902 | 1.322 | 0.586 | 0.940 | 0.815 | 0.799 |
| **RG2** | 1.230 |  | 0.711 | 0.961 | 0.567 | 0.667 | 1.005 | 0.616 | 0.787 | 1.010 |
| **RG3** | 0.943 | 0.711 |  | 0.567 | 0.524 | 0.589 | 0.658 | 0.313 | 0.575 | 0.608 |
| **RG4** | 0.889 | 0.961 | 0.567 |  | 0.775 | 0.778 | 0.725 | 0.667 | 0.602 | 0.785 |
| **RG5** | 0.902 | 0.567 | 0.524 | 0.775 |  | 0.820 | 0.538 | 0.405 | 0.392 | 0.677 |
| **RG6** | 1.322 | 0.667 | 0.589 | 0.778 | 0.820 |  | 1.118 | 0.581 | 0.870 | 1.043 |
| **RG7** | 0.586 | 1.005 | 0.658 | 0.725 | 0.538 | 1.118 |  | 0.659 | 0.394 | 0.513 |
| **RG8** | 0.940 | 0.616 | 0.313 | 0.667 | 0.405 | 0.581 | 0.659 |  | 0.512 | 0.639 |
| **RG9** | 0.815 | 0.787 | 0.575 | 0.602 | 0.392 | 0.870 | 0.394 | 0.512 |  | 0.574 |
| **RG10** | 0.799 | 1.010 | 0.608 | 0.785 | 0.677 | 1.043 | 0.513 | 0.639 | 0.574 |  |

| 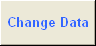   | 1.5 | | --- | | RG3 | RG8 | **Normalisation Factor** | |
| --- | --- | --- | --- | --- | --- |
| 5 DPA,RNA 1 | 6.38E-01 | 7.45E-01 | **1.4240** |  |
| 5 DPA,RNA 2 | 6.18E-01 | 4.23E-01 | **1.0567** |  |
| 5 DPA,RNA 3 | 6.78E-01 | 4.79E-01 | **1.1775** |  |
| 10 DPA,RNA 1 | 1.00E+00 | 1.00E+00 | **2.0652** |  |
| 10 DPA,RNA 2 | 6.65E-01 | 5.16E-01 | **1.2106** |  |
| 10 DPA,RNA 3 | 6.20E-01 | 4.73E-01 | **1.1192** |  |
| 15 DPA,RNA 1 | 7.51E-01 | 5.46E-01 | **1.3224** |  |
| 15 DPA,RNA 2 | 6.05E-01 | 6.44E-01 | **1.2885** |  |
| 15 DPA,RNA 3 | 4.06E-01 | 2.76E-01 | **0.6914** |  |
| 20 DPA,RNA 1 | 4.06E-01 | 2.15E-01 | **0.6098** |  |
| 20 DPA,RNA 2 | 3.70E-01 | 3.49E-01 | **0.7429** |  |
| 20 DPA,RNA 3 | 2.77E-01 | 1.81E-01 | **0.4626** |  |
| 25 DPA,RNA 1 | 5.24E-01 | 3.58E-01 | **0.8946** |  |
| 25 DPA,RNA 2 | 3.37E-01 | 2.61E-01 | **0.6121** |  |
| 25 DPA,RNA 3 | 9.21E-01 | 5.67E-01 | **1.4918** |  |
|  | 0.552975913 | 0.423987874 |  |  |
| **M < 1.5** | **0.313** | **0.313** |  |  |


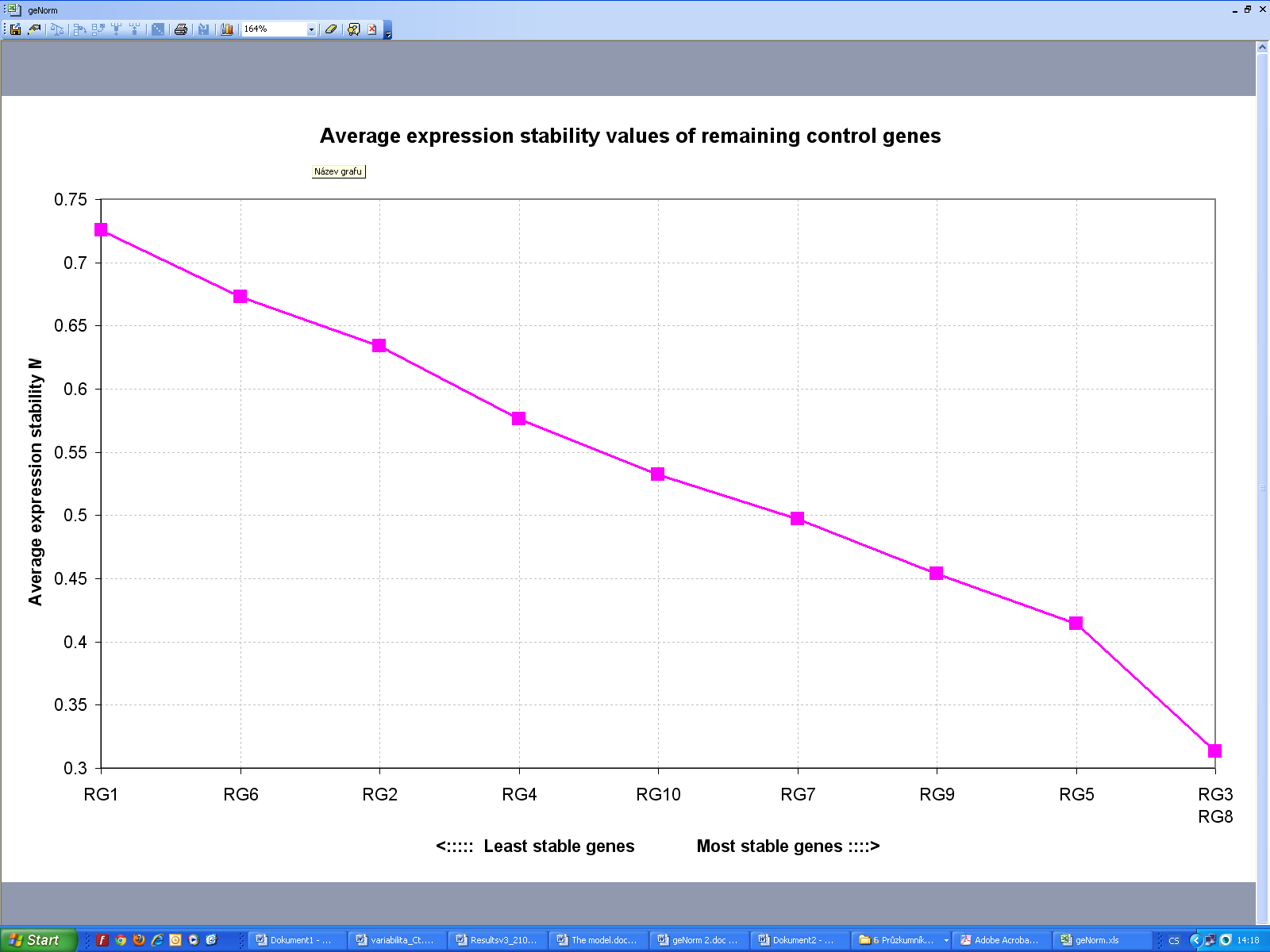


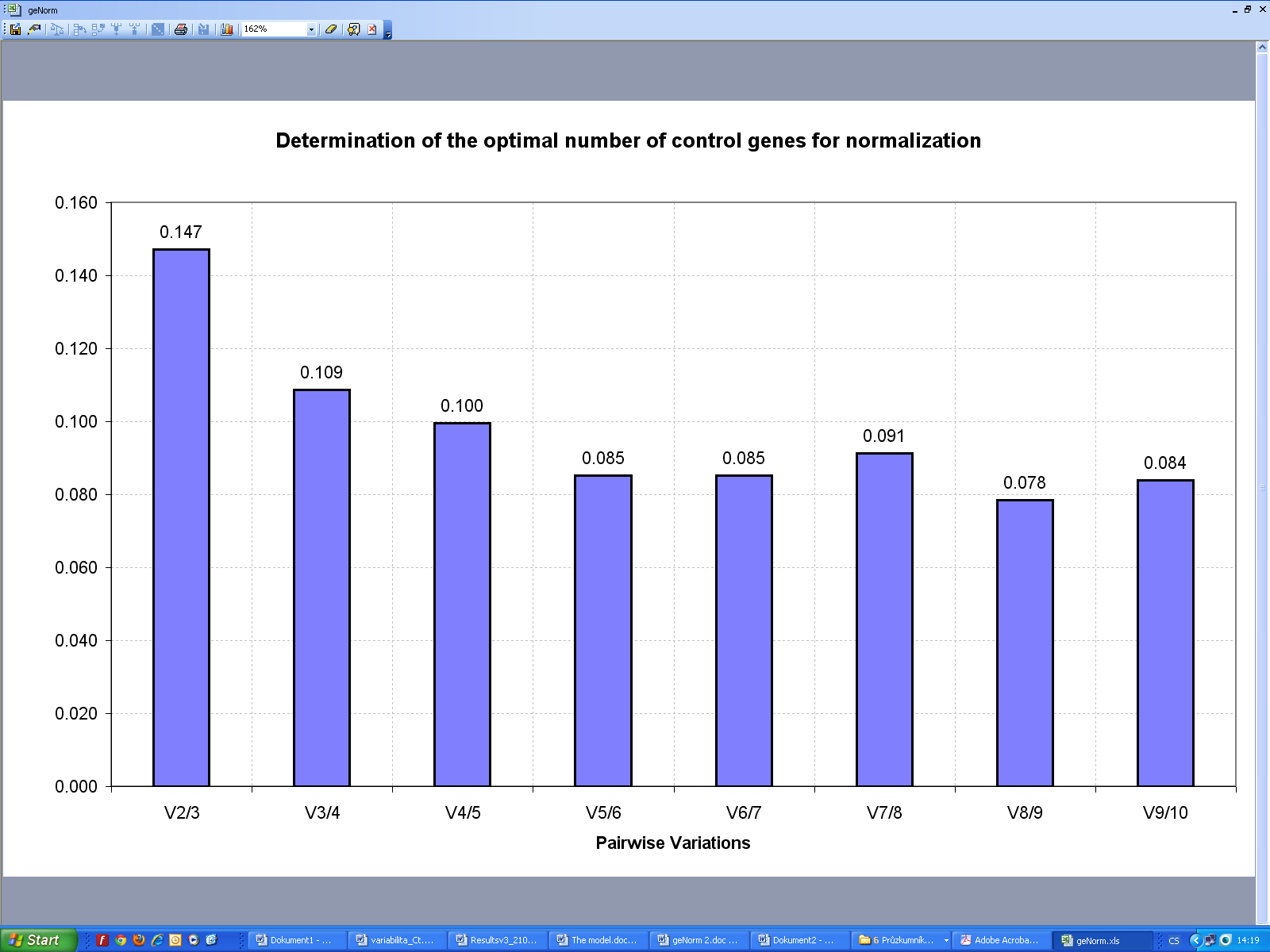

Supplement: Data S1 — Three individual plants per data point were used, from each plant two independent RNA isolations were performed. Ct values were calculated as mean of three different measurement (triplicate analysis). (DOC) [file pone.0041886.s001.doc]
