# Supplementary material for: Validation of the β-amy1 Transcription Profiling Assay and Selection of Reference Genes Suited for a RT-qPCR Assay in Developing Barley Caryopsis
Source: PLoS One. 2012 Jul 31;7(7):e41886. doi: 10.1371/journal.pone.0041886 (PMC3409215; doi:10.1371/journal.pone.0041886)
Supplement: Data S2 — Reported values are the mean of three measures. (DOC) [file pone.0041886.s002.doc]

| **Gene name** | **Stability value** |  |  |  |  |
| --- | --- | --- | --- | --- | --- |
| Rg1 | 0.150 |  |  |  |  |
| Rg2 | 0.119 |  |  |  |  |
| Rg3 | 0.087 |  |  |  |  |
| Rg4 | 0.130 |  |  |  |  |
| Rg5 | 0.082 |  |  |  |  |
| Rg6 | 0.160 |  |  |  |  |
| Rg7 | 0.124 |  |  |  |  |
| Rg8 | 0.095 |  |  |  |  |
| Rg9 | 0.132 |  |  |  |  |
| Rg10 | 0.131 |  |  |  |  |
|  |  |  |  |  |  |
|  |  |  |  |  |  |
| **Best gene** |  |  |  |  | Rg5 |
| **Stability value** |  |  |  |  | 0.082 |
|  |  |  |  |  |  |
| **Best combination of two genes** | |  |  |  |  |
| **Stability value for best combination of two genes** | | |  |  | Rg3 and Rg5 |
|  |  |  |  |  | 0.067 |
| **Intragroup variation** |  |  |  |  |  |
| Group identifier | 1 | 2 | 3 | 4 | 5 |
| Rg1 | 0.090 | 0.060 | 0.017 | 0.002 | 0.040 |
| Rg2 | 0.000 | 0.157 | 0.001 | 0.001 | 0.002 |
| Rg3 | 0.001 | 0.007 | 0.017 | 0.005 | 0.022 |
| Rg4 | 0.000 | 0.080 | 0.004 | 0.001 | 0.059 |
| Rg5 | 0.005 | 0.055 | 0.007 | 0.000 | 0.001 |
| Rg6 | 0.005 | 0.105 | 0.020 | 0.001 | 0.003 |
| Rg7 | 0.015 | 0.124 | 0.007 | 0.000 | 0.000 |
| Rg8 | 0.026 | 0.040 | 0.005 | 0.002 | 0.002 |
| Rg9 | 0.020 | 0.011 | 0.022 | 0.004 | 0.001 |
| Rg10 | 0.036 | 0.116 | 0.010 | 0.001 | 0.017 |
|  |  |  |  |  |  |
|  |  |  |  |  |  |
| **Intergroup variation** |  |  |  |  |  |
| Group identifier | 1 | 2 | 3 | 4 | 5 |
| Rg1 | 0.235 | -0.100 | -0.128 | 0.030 | -0.038 |
| Rg2 | -0.156 | 0.035 | 0.035 | 0.087 | -0.001 |
| Rg3 | -0.057 | -0.022 | 0.034 | 0.007 | 0.039 |
| Rg4 | -0.052 | -0.188 | 0.049 | -0.067 | 0.259 |
| Rg5 | -0.048 | 0.077 | 0.010 | 0.011 | -0.050 |
| Rg6 | -0.210 | -0.090 | 0.112 | 0.068 | 0.120 |
| Rg7 | 0.092 | 0.122 | -0.103 | -0.031 | -0.080 |
| Rg8 | -0.034 | -0.001 | 0.054 | 0.022 | -0.041 |
| Rg9 | 0.063 | 0.168 | -0.029 | -0.125 | -0.077 |
| Rg10 | 0.167 | -0.002 | -0.032 | -0.003 | -0.130 |
